# A Systematic Review of the Reliability and Validity of Behavioural Tests Used to Assess Behavioural Characteristics Important in Working Dogs

**DOI:** 10.3389/fvets.2018.00103

**Published:** 2018-05-25

**Authors:** Karen Brady, Nina Cracknell, Helen Zulch, Daniel Simon Mills

**Affiliations:** ^1^School of Life Sciences, University of Lincoln, Lincoln, United Kingdom; ^2^Defence Science and Technology Laboratory (Dstl), Salisbury, United Kingdom

**Keywords:** affect, behavioural tests, dogs, emotion, personality, reliability, temperament, validity

## Abstract

**Background:**

Working dogs are selected based on predictions from tests that they will be able to perform specific tasks in often challenging environments. However, withdrawal from service in working dogs is still a big problem, bringing into question the reliability of the selection tests used to make these predictions.

**Methods:**

A systematic review was undertaken aimed at bringing together available information on the reliability and predictive validity of the assessment of behavioural characteristics used with working dogs to establish the quality of selection tests currently available for use to predict success in working dogs.

**Results:**

The search procedures resulted in 16 papers meeting the criteria for inclusion. A large range of behaviour tests and parameters were used in the identified papers, and so behaviour tests and their underpinning constructs were grouped on the basis of their relationship with positive core affect (willingness to work, human-directed social behaviour, object-directed play tendencies) and negative core affect (human-directed aggression, approach withdrawal tendencies, sensitivity to aversives). We then examined the papers for reports of inter-rater reliability, within-session intra-rater reliability, test-retest validity and predictive validity.

**Conclusions:**

The review revealed a widespread lack of information relating to the reliability and validity of measures to assess behaviour and inconsistencies in terminologies, study parameters and indices of success. There is a need to standardise the reporting of these aspects of behavioural tests in order to improve the knowledge base of what characteristics are predictive of optimal performance in working dog roles, improving selection processes and reducing working dog redundancy. We suggest the use of a framework based on explaining the direct or indirect relationship of the test with core affect.

## 1. Introduction

### 1.1 Rationale

Animal behavioural tests can be defined as standardised experimental situations where stimuli serve to elicit behaviour that is statistically compared with that of other individuals in the same situation, with the aim of classifying the tested animal (1). Dog behavioural tests have been developed and applied across a wide range of areas, including for genetic and breeding evaluation (2, 3), for assessment of behavioural development (4), and learning abilities (5), as well as for predicting outcomes such as likelihood of shelter adoption (6). Here, we focus on the role of behavioural tests as predictors of success (i.e., desirable performance during/after training and in a working role) for “working dogs”, herein defined as a dog that has or is being selected for a working role which is either associated with assistance work, protection work, or detection work, and is regulated and certified for such work. With growing recognition of the value of working dogs to assist individuals with physical, emotional and developmental issues (e.g., 7; 8, 9), and the importance of military working dogs in the current global political climate (10–12), it has perhaps never been more important to evaluate the quality of procedures used to predict the success of these working animals, in terms of their ability to perform optimally in their specified role.

Although the training and sourcing of military and assistance dogs is associated with high financial costs (13, 14), dogs work in many valuable roles, contributing to industry development and performance (15) and the benefits of assistance dogs are associated with considerable economic savings, in terms of reduced reliance of mainstream support services, such as the NHS (16, 17). Nonetheless, it is suggested that across the sectors, on average only 50% of working dogs become fully operational (18), (19–23). Furthermore, a predominant theme in the working dog literature is that some dogs perform better at their assigned duties than others, with behavioural characteristics rather than sensory sensitivities or morphological differences largely accounting for the level of success achieved [e.g., (21, 24–26)]. Not only does this affect the economic value of the work achieved, but also the perception of the public relating to the importance of maintaining working dogs in society (27, 28).

There are several methods for assessing dog behaviour, including a range of experimental behavioural tests (e.g., observations of the dog’s behaviour in a novel situation; (29) and owner or handler completed questionnaires (e.g., Positive and Negative Activation Scale; PANAS: (30). Behavioural observation tests have been used to assess a range of factors that may be important in working dogs, variously described as “character” (31), “personality” (32) and “temperament” (e.g., 33; 34). A key principle behind many behaviour test methods is behavioural observation of the dog within a situation to evaluate (a) the presence or absence of specific postures or behaviour (e.g., biting) to quantify a behavioural tendency (e.g., aggressivity) (e.g., (35), or (b) subjective ratings of specific behaviour (e.g., calmness) within the test situation, made by trained or familiar observers on a Likert-scale (e.g., 1 = not at all calm; 6 = very calm) (e.g., (36). There is no consensus on the distinction of the terms used relating to the profiles of behaviour produced by these tests or questionnaires, and they are sometimes used interchangeably. For example, the Canine Behavioral Assessment and Research Questionnaire (37) is referred to by the authors as a behaviour and temperament assessment, but it describes the aggregation of context specific behaviour; we therefore suggest it might be best referred to as an assessment of “character” or “behaviour profile”, with the term “personality” 36 reserved for those instruments designed specifically to describe the more general biologically-based traits underpinning individual differences (e.g., Monash Dog Personality Questionnaire; (36)); and the term “temperament” be reserved for instruments focused on the more limited construct of affect and its regulation (e.g., Dog Impulsivity Assessment Scale; DIAS: (38). This distinction may help to clarify thinking about what is being assessed and what is most valuable in a given situation.

Although questionnaires can reduce the need to implement behaviour tests that can be time consuming, and can assess behaviour over a wide range of situations, it is not always possible to source an individual that has sufficient knowledge about the dog to reliably complete the items. This may be particularly true in the case of working dog assessments, which are often done at an early age, by unfamiliar (39) and familiar handlers [e.g., (7, 40)]. Furthermore, behavioural tests arguably provide a more objective assessment of the dog’s behaviour, rather than relying on personal memories and perceptions of handlers/owners who may be biased by the bond with the animal being assessed.

The value of behavioural observation tests should be determined from their reliability and validity (41). Specifically, in working dogs, it has been proposed that it is important that behavioural tests are judged on three key criteria (22):

1. Inter-rater reliability: the extent to which different observers describe the same individual the same way.
2. Test-retest reliability: the extent to which behavioural tests identify characteristics that are stable across time and context; individuals’ scores should generalise across time and condition.
3. Predictive validity: in the case of working dogs, the trait should also be relevant to some aspect of performance and so be predictive of success perhaps in terms of certification and/or long-term performance in the field.

Identifying which behavioural tests are reliable and valid procedures for the assessment of working dogs may not only improve the work accomplished by these dogs (i.e., by selecting optimal performers), but also reduce the associated time and cost implications with training unsuccessful dogs who may not demonstrate the desired behavioural characteristics which are essential for successful performance.

### 1.2 Objectives

The objectives of this systematic review were therefore:

- To identify the range of behavioural tests used for assessing behavioural characteristics in working dogs, that are described in the peer-reviewed scientific literature and assess the quality of these tests.
- To synthesise the available evidence from the scientific literature relating to the validity and reliability of tests used to examine basic biologically based traits that may be important to the success of working dogs.

### 1.3 Research Question

The research question addressed by undertaking this systematic review of the scientific literature available was: To what extent are the range of behavioural tests used for assessing biologically-based traits for working dogs reliable and valid?

## 2. Methods

### 2.1. Study Design

**Figure 1:**
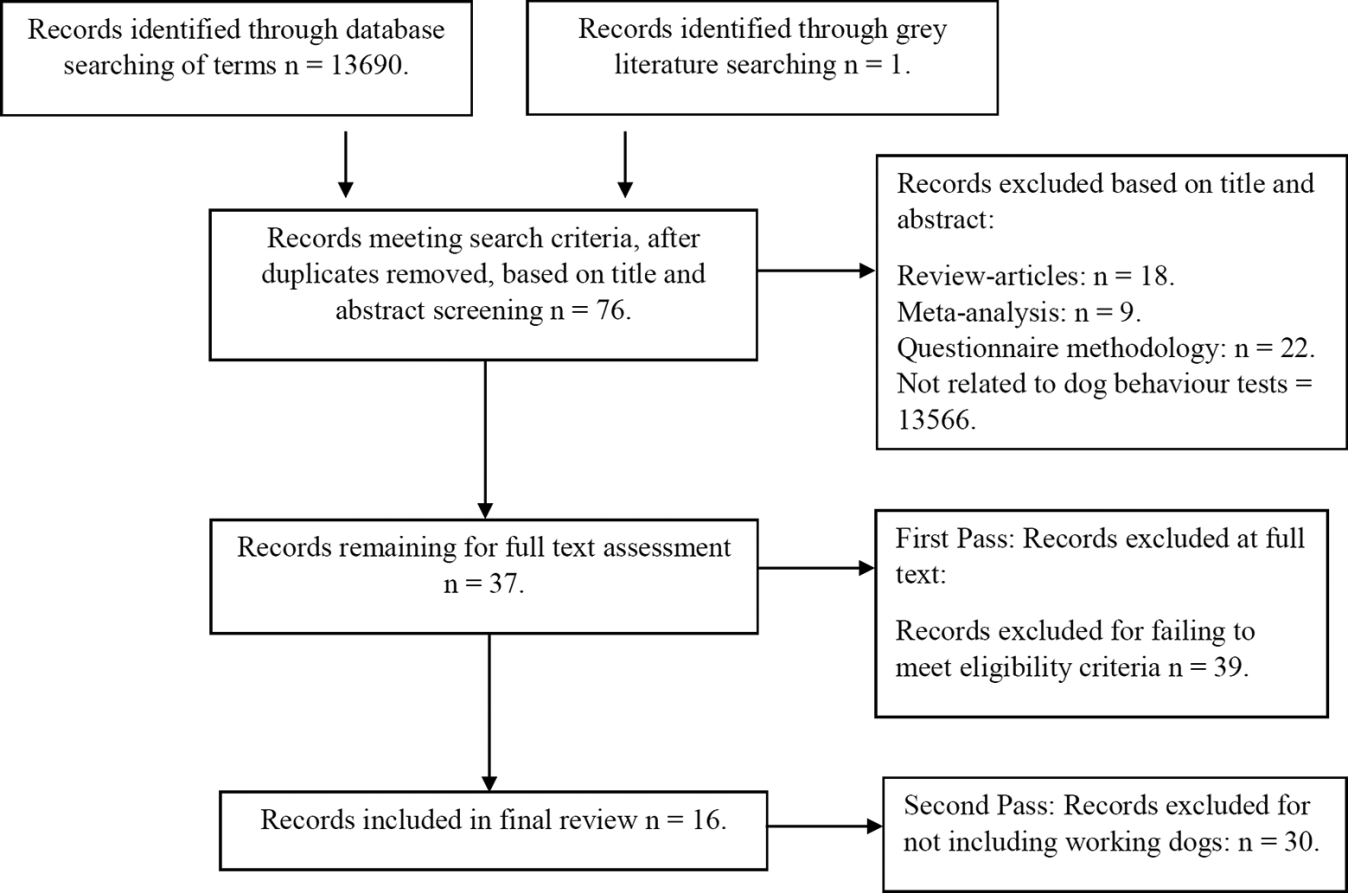
Preferred Reporting Items for Systematic Reviews and Meta-Analysis (PRISMA), flow chart completed for the current study

The Preferred Reporting of Items for Systematic Reviews and Meta-Analyses (PRISMA) guidelines were adhered to for this review (42) (Figure 1).

### 2.2. Participants, Interventions and Comparators

Participants for this study were taken from the scientific peer-reviewed literature and were dogs assessed by one or more behavioural tests that were relevant to the potential assessment of working (including military, service and assistance type) dogs. There were no comparators for this review.

### 2.3. Systematic Review Protocol

The inclusion criteria for selection of articles were:

1. Articles written in English,
2. Literature that reports observable behaviour tests (as opposed to invasive or physiological tests or questionnaires) used to assess dog behaviour characteristics relevant to working dogs,
3. Articles accessible via direct download or contact with the authors.

We did not make exclusions based on dog characteristics (i.e., age, breed, pet or type of working dog status) or test parameters. We stipulated that the studies should include a working dog (or potential working dog) sample. Reviews and meta-analyses were excluded. Questionnaire based studies were also excluded, since these did not satisfy the requirements of a “behavioural observation test”. Studies which did not assess factors which may relate to working dog performance were excluded (e.g., papers purely focusing on the heritability of traits).

### 2.4. Search Strategy

Table 1 contains the list of search terms used. Search terms were decided following expert consultation with established researchers in the field and through evaluation of common terms used in titles and abstracts of papers known to the researchers. At each stage of the review process, a selection of articles were cross-checked by another researcher to ensure agreement on inclusion and exclusion decisions. Full text articles for all papers were sourced electronically, or through direct contact with authors.

**Table 1:** Search terms used in the literature search.

	**Scopus**	**PubMed**	**Science Direct**
Assistance dog behaviour(s)	70	1	5
Assistance dog performance	5	0	2
Assistance dog temperament	7	1	5
Dog behavioural test(s)	1,144	19	14
Dog performance	1,1000	7	8
Dog personality	398	11	10
Ideal working dog	14	0	2
Military dog behaviour(s)	57	1	8
Military dog performance	39	1	6
Military dog selection	15	0	8
Military dog temperament	7	3	8
Police dog behaviour(s)	58	1	12
Police dog performance	29	0	7
Police dog selection	14	0	12
Police dog temperament	5	1	12
Predictability of dog behaviour(s)	24	1	2
Predictability of working dog behaviour(s)	4	0	2
Service dog selection test(s)	18	1	8
Service dog temperament test(s)	8	1	9
Working dog assessment(s)	181	0	12
Working dog performance	254	4	8
Working dog personality	33	1	10
Working dog selection test(s)	31	1	13
Working dog temperament test(s)	22	1	14

### 2.5. Data Sources, Study Selection and Data Extraction

Literature searches were conducted in the following electronic databases: PubMed, Scopus, and Science Direct, from their first year of reporting up to the end of 9^th^ March 2018. Papers were reviewed to identify the range of behavioural characteristics assessed in the selected literature and to categorise each behaviour into a thematic group, relating to underlying traits. Traits are typically inferred from behavioural tests and different tests were expected to label similar traits using different terminologies. To manage this, it was decided to classify the proposed traits assessed by the behavioural tests around a framework extending from their direct or indirect relationship with core affect (positive versus negative emotional states), see Table 2. Positive emotional states were characterised as those related to sensitivity to salient positive qualities in the environment, as observed through behaviour such as human, or object-directed play tendencies. Negative emotional states were characterised by tests relating to sensitivity to potentially aversive qualities in the environment, as observed through behaviour such as human-directed aggression and approach-withdrawal tendencies.

**Table 2:** Categorisation of behaviour assessed within the working dog selection literature to create thematic groups grounded in a biological basis for behaviour.

Core Affect	Behavioural Characteristic	Examples of Terms Used	Examples of Test Parameter
Positive emotional state	Willingness to work	Search focus, motivation	Searching for an object without interruption (22, 23) / hunt drive (43).Speed and hesitation with obstacle crossing (23, 44, 45).Distraction behaviour shown when another dog passes by (20).Ratings of willingness to return a ball/object (34, 43), trainability (25, 46). and willingness to chase/follow light spots (25).
	Human-directed social behaviour	Greeting behaviour, approach to strangers	Ratings of willingness to greet a stranger (34, 45, 47).Body posture / behaviour during approach, petting/examination (44, 46).
	Object-directed play	Toy play, chase	Behaviour and vocalisations during toy play (22).Time to release toy (22) and latency to catch toy (20).Intensity and interest in toy/tug-of-war (45, 47).Immediate reaction to toy (investigate first or start to play) (34).Responsiveness to toy versus assessor (44).
Negative emotional state	Human-directed aggression	Defence drive, stranger-directed aggression	Posture, behaviour (22) and vocalisations towards tester (22) /strangers (25).Speed to bite and force of bite to tester (22)Level of aggressive response when provoked (23), startled (47), or approached (11).
	Approach-withdrawal	Investigation-exploration	Exploratory behaviour when startled, by visual or acoustic stimuli (11, 39, 47) and when in a novel environment (48), or with novel objects (49, 46).
	Sensitivity to aversives	Noise sensitivity, gunshot tests, sudden appearance tests	Steadiness / sureness during gun tests, marking of behavioural postures (22, 23, 50).Avoidance reactions during gun tests (47).Startle reaction to visual and acoustic stimuli (51, 11, 44, 45, 47, 48, 50, 52, 53)Latency to recover from noise (20, 39).

To assess the reliability and validity of the behavioural tests, we requested further statistical information on the reliability of the reported behaviour tests, from corresponding authors of all the papers.

### 2.6. Data Analysis

Data obtained in the papers relating to inter-rater, intra-rater (within session), test-retest reliability and predictive validity were pooled at a behavioural trait level (as determined in the first stage of the analysis, described above). This information was then examined to give an indication of the overall quality of selection tests in measuring specific behavioural traits potentially relevant to a variety of working dogs and to evaluate if these traits were predictive of successful performance in the field.

## 3. Results

### 3.1. Flow Diagram of the Studies Retrieved for the Review

A flow diagram summarising the outcome of the retrieval process at each stage of the review is provided in Figure 1. Papers rejected in the second pass analyses are reported in Data Sheet S1.

### 3.2. Study Selection and Characteristics

The initial literature search, using the terms specified in Table 1, produced 13,690 hits, with an additional reference obtained from grey literature searches (*n* = 13691). After title and abstract screening, records which appeared to match the inclusion criteria were included in the first pass full text analysis (*n* = 76). Upon completion of the first pass 39 records were further excluded for failing to meet the eligibility criteria. The remaining papers (*n* = 37) were included in a second pass analysis, consideration for inclusion was discussed by authors and an independent team member (see Data Sheet S1), leaving 16 papers for inclusion in the final review (see Figure 1). The remaining 16 papers were fully assessed in accordance with the two stages described in the methods above. In response to our request for further data from corresponding authors, one author declined to comment as this was being used for future work and another indicated there was no further information. The rest did not respond, or the corresponding author’s email was no longer active.

The data relating to the articles examined and their classification are summarised in Table 2. A range of behavioural tests and parameters were used to measure positive emotional states, such as body posture during human contact (e.g., (44), time to release toy (22) and latency to catch toy (20). There was a large range of behaviour tests and parameters used to assess negative emotional states, such as level of aggressive response when provoked (23), startled (47), or approached (11) and behaviour during gun tests (22, 23).

### 3.3. Synthesised Findings

Details of our assessment of the quality metrics of each trait are given in the supplementary information in Table S1A and Table S2, but summarised below. We generally use the terminology used by the authors, but caution is necessary to avoid unwarranted generalisation of the terms used in a test report to any underlying biologically-based trait of the same name (e.g., boldness as a short hand of the outcome of a particular test, and the true trait of boldness).

#### Inter-Rater Reliability

There was little reporting of inter-rater reliability statistics; agreement across two or more independent observers was only reported in four of the 16 papers included in the review (11, 22, 43, 44). Three papers discussing behaviour related to positive affect touched upon inter-rater reliability (22, 43, 44). Two of these papers reported quantitative statistics - in the form of significant correlations between raters’ scoring behaviour surrounding “willingness to work” and “object directed play” (22, 43). For behaviour related to negative affect, three papers considered inter-rater reliability (11, 22, 43). These papers reported quantitative statistics to support the statement that ratings on sensitivity to aversives, approach-withdrawal and human-directed aggression were reliable across raters (11, 22, 43).

#### Intra-Rater Reliability

Fewer studies reported intra-rater reliability statistics; agreement within a single rater’s scores within a session was only reported in three of the 16 papers included in the review (22, 44, 46). These papers reported statistics for behaviour relating to both positive and negative core affect. One study claimed 90% agreement between time points (44), and the other "good" intra-class correlation coefficients (22), but only one reported statistical tests to support these claims directly (46).

#### Test-Retest

Test-retest statistics were reported by four papers (11, 22, 43, 46). Test-retest reliability for positive affect related behaviour (22, 43, 46), were mixed. Assessing behaviour surrounding willingness to work, Sinn et al. (22) reported significant correlations across and between three-time points for some behaviour (object focus, sharpness, and human focus), but less reliable correlations for other behaviour (search focus). McGarrity et al (43) reported low (54) Intra-Class Coefficients (ICC) over time for behaviours relating to willingness to work (≤0.28: including hunt drive, search performance and search aptitude) and object directed play (≤0.13, including dominant possession and independent possession. Similarly, Harvey et al. (46) reported poor correlations for human-directed social behaviour (0.33–0.45) and object-directed play (0.39–0.46).

For the papers pertaining to negative affect related behaviour, four reported test-retest statistics (11, 22, 43, 46). One of these papers reported aggregate scores across sub-tests, therefore it is not possible to directly associated the data with specific behavioural aspects, nonetheless this paper reported high coefficients across time points (α = 0.89) (11). Two papers reported test-retest correlations for behaviour specifically associated with human-directed aggression, with one study reporting significant correlations (22) and another reporting average correlations (46). Three papers reported test-retest statistics for behaviour relating specifically to sensitivity to aversives (22, 43, 46). Findings were mixed even within a single study, with evidence of moderate correlations in some tests, but not others (46). In general, test-retest statistics on behaviours relating to sensitivity to aversives were low (22, 43). It should be considered, that when testing test-retest reliability particularly with young animals, that the gap between to the testing times may influence the results and that a lack of correlation does not imply a lack of test-retest reliability. Instead, it is important to consider normative change; in that individuals develop similarly so that they maintain their rank order between testing times (e.g., (55).

#### Predictive Validity

Behaviour associated with willingness to work predicted success in: (i) guide dog training (retrieve response to stimuli: (44, 52); distraction and passive test success (20); response to commands; (46), (ii) police/military dog certification/efficiency (search focus and sharpness (22); retrieve performance at eight-weeks (23); decreased scores on the factor ‘movement’ (45); higher scores on trainability, hyperactivity and chasing/following lights: (25) and (iii) odour detection dogs (hunt drive: (43). However, Wilsson and Sundgren (34) found limited utility in their behavioural test, which assessed behaviour relating to willingness to work, for predicting future service dog performance.

Some behaviour associated with human-directed social behaviour were associated with success in guide dog training (stroking response to assessor (44); not displaying low body posture during greeting; (46), better performance in working dog trials (sociability towards strangers: (47), police dog efficiency tests (factor for movement, incorporating behaviour towards a person: (45) and greater cooperation at maturation (34). However, two studies which investigated human-directed social behaviour, showed a lack of evidence for predictive validity in terms of both guide dog work (52) explored this behaviour but did not report any positive or negative predictive effects) and service dog performance in a retrieval task (48).

Behaviour associated with object-directed play did not reliably significantly predict success in guide dog training with evidence of no predictive effects in two studies (squirrel-response to stimuli (44); latency to catch: (20), but reports of a predictive effect in one study (playing with a tea-towel: (46). Additionally, object-directed play did not predict service dog performance (34), but did predict success in police dog efficiency tests (attitude to predation, including retrieval and tug of war: (45), performance in working dog trials (boldness, related to playfulness: (47) and odour detection dogs (dominant possession: (43)).

Behaviour associated with human-directed aggression predicted police dog (23) and military dog (25) efficiency, but no other reports of human-directed aggression predicting future working performance were mentioned. One of the potential issues with assessing aggression which could account for a lack of predictability, is that it may not be a personality trait that can be predicted from the limited range of contexts possible in a field test. Aggressive behaviour is often a response to fear, frustration and/or pain, and the tendency to use aggression may differ between individuals depending on specific context. Many behaviour tests focus on aggression in response to fear eliciting stimuli, and so the tests will not be predictive of the behaviour, even if used more generally in other situations, such as in relation to reward denial (a form of frustration).

There was conflicting evidence as to whether behaviour associated with sensitivity to aversives predicted success in guide dog training. Reports of latency to recover from noise predicted guide dog success when tested at 12 and 14 months (20). Additionally, latency to sit during passive and noise tests predicted success when tested at 13–17 months (39). However, behavioural responses (e.g., shaking) to noise at 6–8 weeks did not (44) and there was no evidence that sensitivity to aversives predicted success in general service dog work (48). Similarly, gunshot sensitivity did not predict adult police dog efficiency whereas startle test responses were higher in those who became police dogs than those who did not (23). In contrast, a more positive/less fearful response to aversives predicted a lower probability of passing police/military dog training (response to a noise (45); response to non-social fears: (25)), whereas dogs who scored high in boldness (related here to fear) performed significantly better in working dog trials (47) and dogs who were selected for military work scored higher on ambivalent and overt fear than non-selected dogs (53). Two further studies reported predictive validity of their tests for sensitivity to aversives, but not in terms of predicting ultimate working dog success. One of these studies reported that, for potential guide dogs, sound sensitivity ratings positively correlated with fear ratings and reaction to the pinch test correlated with submission ratings (52). The other study reported that the Emotional Reactivity Test (ERT) significantly increased salivary cortisol and plasma in the dogs, suggesting the test can be used to identify dogs with a low threshold for emotional reactivity (11). Whilst these studies do not tell us much about the potential desirability of certain traits they do highlight the validity of specific tests which may be considered for inclusion in the development of future protocols.

There was little evidence of applying approach-withdrawal behaviour to predicting working dog success. Two studies explored these tendencies in relation to guide dog performance (39, 46) and one in relation to service dog performance (48) but failed to find a signification relationship. However, in relation to military dog performance, Foyer et al. (53) reported that approved dogs had higher scores on active avoidance than non-approved dogs, indicating some possible predictive validity for tests which assess this characteristic.

### 3.4. Risk of Bias

This review considers only tests published in peer reviewed English literature, and so does not consider the full range of tests that may be in use and any supporting documentation produced in the course of their development, which may further evidence the quality of the test. Nonetheless, by focusing on the peer reviewed literature, we would argue that we are focusing on the tests with the most rigorous data available. Thus if there is any bias, it is perhaps a skew towards an overestimation of the quality metrics available. We do not believe our exclusion criteria have introduced a significant bias to our interpretation of the data.

## 4. Discussion

### 4.1. Summary of Main Findings

It was evident that a large range of tests are used to assess the behaviour of dogs (e.g., gun fire, sudden appearance test, obstacle courses, stranger approach, toy/kong tests) with a range of parameters used to indicate performance (e.g., subjective rating scores of body postures, scores of vocalisations, time taken to achieve target). We observed that some of these tests not only assessed similar traits, but also labelled similar traits using different terminologies. If we are to make accurate, direct, comparisons of the validity of behavioural tests and make inferences on the importance of specific traits for working dog performance, researchers need to be aware of the importance of using consistent terminologies. We therefore grouped behavioural tests according to the putative underlying traits they assessed relating to either positive affect (willingness to work, object-directed play, human directed social behaviour) or negative affect (human directed aggression, sensitivity to aversives, approach withdrawal).

With the aim of identifying the reliability and validity of tests of potential value for predicting the performance of working dogs, we used standard statistics relating to these for the behavioural tests available from the literature. Good quality reporting of inter-rater and intra-rater reliability statistics was notably lacking. For behaviour associated with positive affect, only three studies reported significant correlation between raters (22, 43, 46), but one of these papers (22) failed to report Cohen’s Kappa (56), or alternative coefficient statistics (57). For behaviour associated with negative affect behaviour, four papers were identified as providing supportive statistics (11, 22, 43, 46). Only one paper (46) reported Intra-class Correlation Coefficients (ICC) for within rater reliability assessment (58). This leaves doubt over the objectivity of the data obtained from any and all of these tests, since without showing some consistency between or within observers, this should not be assumed. We recommend that reporting of such metrics be an essential requirement for scientific publication in future.

The importance of evaluating test-retest reliability results is further highlighted by our findings; with only four papers reporting these statistics (11, 22, 43, 46). It is important to consider that test-retest reliability results are likely to be affected by the study design (i.e., delay between test-retest) as well as the reliability of the behaviour test. It is also important that tests report correlations as well as statistical tests of differences between values over time, since it is possible to have good correlation without repeatable results (i.e., the intercept of the correlation is not through zero), and any consistent difference between tests needs to be known so it can potentially be corrected for.

Nonetheless, there was some evidence for test-retest reliability for both aspects of negative affect (sensitivity to aversives (11); human-directed aggressive behaviour (22) and positive affect (object-directed play (22). When considering test-retest reliability, distinction between a behaviour and a trait is particularly important (59). Traits are typically inferred from a behaviour which has been observed across situations, whereas specific behaviour may disappear over time due to changes such as behavioural habituation, rather than unreliability of the test *per se*, highlighting the importance of considering features of the test, such as predictive validity.

Positive affect behaviour related to performance in guide dog roles (willingness to work, human-directed social behaviour; (20, 44, 46), police/military dog work (willingness to work, human-directed social behaviour and object-directed play; (45), (25, 53), in working dog trials (human-directed social behaviour, object-directed play (47)) and for odour detection dogs (willingness to work, object-directed play (43)). However, it is important to note that a significant result *per se* may not be sufficient for the test to be valuable, and closer inspection of metrics such as the variance around the correlation are of importance. An additional important point to consider is that predictive validity (in terms of long-term performance) should be compared against concurrent validity (i.e., an outcome assessed at the same time as the behaviour test), particularly in young dogs, since a lack of predictive validity may not be due to a weakness of the test, but rather reflect the dog’s development (46).

There was less consensus across the papers on whether negative affect behaviour predicted working dog success. Only one report indicated that human-directed aggression predicted police dog efficiency (23), and three papers reported the predictive value of approach-withdrawal tendencies (odour detection dogs (43), military dogs (53), guide dogs (46)). There were conflicting results with regard to sensitivity to aversives. Indeed, whereas gunshot sensitivity did not predict police dog success, startle response did (23), and the initial startle may be a better predictor of general autonomic sensitivity, since the response beyond this will depend on higher level appraisal of coping ability. Furthermore, a more positive response to noise (less fear, more exploratory behaviour) at 7 weeks old was associated with a lower likelihood of passing police dog certification (45). This highlights the importance of consistency of test characteristics and requirements when assessing behavioural traits. Similarly, with regard to guide dogs, latency to recover from an aversive stimulus did not predict later guide dog success (20), whereas behavioural response to an aversive stimulus did (44, 46), emphasising the importance of using consistent parameters when comparing performance across tests. However, it could also be that these contrasting results reflect age-related developmental differences in the dogs, with Asher et al (44) working with younger dogs (6–8 weeks) than Batt et al (20) (>6 months). Nonetheless, this would stand in contrast to reports which claim that important guide dog traits can be measured more reliably in older puppies (>14 months; (20). Additionally, there is considerable disparity in the sample size between these studies [e.g., (44), *n* = 587; (20), *n* = 43]; it is plausible that Batt et al. (20) lacked statistical power to observe potentially significant effects relating to responses to aversive stimuli. It should also be considered that the simple pass/fail criteria used to determine guide dog success ignores the disparity of outcomes which may be associated with successful performance as a guide dog, which limits the test specificity and accuracy.

Regardless of the nature of a dog’s job, temperament and personality are important for a dog to fulfil its role and certain traits were relatively consistently referred to and putatively measured in the literature despite differences in working dog roles. The most commonly measured temperament trait related to sensitivity to aversives, with the majority of behaviour tests aiming to examine fearful type responses to potentially threatening stimuli. Different traits are important to different extents depending on the role of the dog, for example tendency to show aggression may be desirable in a military working dog, but undesirable in a guide dog; nevertheless knowing about it is important to both types of working dog. It is therefore important to identify the optimal behavioural phenotype for certain working roles (59), to enable better selection of dogs for working positions which could reduce dropout rates, increase success of certification and also improve the dog’s welfare, as certain dogs may not be psychologically robust enough, or suitably predisposed, for certain working roles.

Researchers use different indices to validate their tests, along with different parameters and metrics to score specific behaviour, there is an inconsistency in categorising behaviour, with different terminologies used to refer to the same behaviour (60), and different authors may propose their own frameworks for conceptualising these traits. We have suggested one based on core affect in specific subcategorical contexts, as it is more descriptive and generic and may encompass others which are more specific. For example the categories proposed within McGarrity et al’s, framework (2015), include traits such as “sociability’, which in this review would fall into the Human Directed Social Behaviour category, ‘activity' and ‘trainability/responsiveness’, which would both fall into the Willingness to Work category, while ‘boldness/self-assuredness” and “exploration” would fall into the Approach Withdrawal Tendencies category; all of which would be under the broader framework of Positive Activation. Likewise, a number of categories proposed by McGarrity et al, (61), such as “fearfulness/nervousness’, ‘reactivity’, and ‘submissiveness” fall into our Sensitivity to Aversives category and “aggressiveness” falls into our Human Directed Aggressive Behaviour category; both of which come under the broader feature of Negative Activation. Further subcategories can be added to our proposed framework if required, but by grouping similar behaviour into thematic groups, based on these more general classes of trait, we provide a framework for synthesising the data from the diverse tests reported on in the literature, and encourage future researchers and those responsible for developing tests in practice to consider how their work fits within this framework. In particular it is important to distinguish between the goal of developing a test to evaluate a specific response of interest (e.g., fear of gunshot) and a more general trait (e.g., fearfulness). In the case of the latter, the trait should be put within a sound biological conceptual framework (e.g., core affect, impulsivity etc). By grouping terminologies into thematic groups based on their relationship with underlying core affect (positive and negative), we have identified that reliable behavioural tests for assessing positive affect may be of particular interest to a range of service dog providers, given evidence of their validity for predicting success of those in guide dog roles (willingness to work, human-directed social behaviour), police dog work (willingness to work, human-directed social behaviour and object-directed play) and working trial dog (human-directed social behaviour, object-directed play). The predictive validity of negative affect behaviour is less clear, although anecdotally we believe many organisations seem to have a particular focus on assessing this, through trying to evaluate concerns over fearfulness. We suspect this may be due, at least in part, to a mistaken belief that confidence comes from a lack of fearfulness (62). Our data suggest, that perhaps there is a need for a cultural shift to focus on assessing confidence *per se,* rather than timidity, and to recognise that these are different traits (in line with descriptions of core affect, and scales such as the Positive and Negative Activation Scale for dogs; PANAS, (30), and not opposite ends of the same trait. Weak conceptual frameworks alongside inconsistent use of terminology, test parameters and indices of success are likely to reduce the predictive value of tests for assessing future working dog performance.

### 4.2. Limitations

While the focus of this review was on gathering information about the available behavioural tests, as this form of assessment is the most common way of assessing working dogs, it is important to note that other methods can be used. Physiological measures may reveal biological responses to situations and these can be correlated with behaviour tests [e.g., (11, 63)], providing convergent validity for the tests, but the presence of this evidence was not considered in this review. In some situations it may not be possible to use a behaviour assessment, for example if a dog is physically impaired, or there is a lack of space, time, or access for the dogs under assessment. In such circumstances questionnaire data based on experience with the dog over a prolonged time can be used to get behavioural information from the owner, handler or trainer to identify the temperament or ability of a dog. Examples include the Positive and Negative Activation Scale; PANAS (30) and the Dog Impulsivity Assessment Scale; DIAS (38). However, questionnaires require someone to have sufficient knowledge of the dog, as well as relying on receiving accurate and honest information from those completing it. This may be a particular concern within the working dog sector, given the high value of working stock. Although behavioural tests are usually the favoured form of temperament assessment, a combination of behavioural, physiological and questionnaire measures brings convergent validity to the process, strengthens any conclusions and allows assessment according to the feasible means available in a given context.

This review was limited by the restricted availability of original data sources that might have helped us assess the reliability and validity of the tests. We suggest that such data be made available as a matter of routine either within publications or within accessible electronic repositories, if some restriction is required. Although our search strategy means other relevant publications may exist, we believe the papers presented in this review provide a reasonable representation of the types of tests in use for which data are available, and if anything overestimate the reporting and knowledge of quality metrics, since they tend to be well-cited. We also recognise that this systematic review, like any review is limited by the search strategy, which is never entirely objective and so the results may not be comprehensive and could be biased by papers not revealed in the searches. However, critical features associated with the scientific quality of systematic reviews are that they are replicable and that the conclusions are based on the evidence revealed by the search. Using this approach we believe we can suggest useful insights into both past and future work, through the framework that was revealed by the thematic analysis.

### 4.3 Conclusions

In conclusion, this review indicates that we are still not addressing concerns over the lack of standardisation amongst research on dog behavioural tests [e.g., (41, 64)]. We suggest test developers clearly focus on whether they need a test which seeks to assess specific behaviour (which we recommend be referred to as tests of elements of “character”), or more general biologically-based traits in line with human personality research (which we recommend be referred to as “personality” tests). The term “temperament” test should perhaps be reserved for a subset of personality tests, which seek to assess traits relating to emotionality, rather than more cognitive processes associated with individual differences (such as sociality) although we recognise the two clearly interact to define the individual’s behavioural tendencies. Nonetheless, we suggest there is value in considering temperament in terms of the regulation and expression of core affect. Conceptualising the optimal phenotype of a working dog in a given context in terms of the relative importance of sensitivity to both rewards and aversives provides a biologically-based framework around which the results of diverse tests can be evaluated. In this regard, it seems there may be particular value in establishing the full validity of methods aimed assessing positive affect in dogs, since the data to date, suggest this may have predictive validity for working success.

## Author Contributions

DM, HZ, NC and KB. Designed the study KB. Executed the initial search KB, DM, HZ, and NC. Reviewed data generated by search KB, DM, HZ, and NC. Contributed to the writing of the paper.

## Conflict of interest statement

The authors declare that the research was conducted in the absence of any commercial or financial relationships that could be construed as a potential conflict of interest.
