# Supplementary material for: A Systematic Review of the Reliability and Validity of Behavioural Tests Used to Assess Behavioural Characteristics Important in Working Dogs
Source: Front Vet Sci. 2018 May 25;5:103. doi: 10.3389/fvets.2018.00103 (PMC5982092; doi:10.3389/fvets.2018.00103)
Supplement: Supplementary file 1 [file Table1.docx]

| **First Author (Date)** | **Working Dog Purpose** | **Inter-Rater** | **Intra-Rater** | **Test-Retest** | **Predictive Validity** | | | | | |
| --- | --- | --- | --- | --- | --- | --- | --- | --- | --- | --- |
|  |  |  |  |  | **Willingness to Work** | **Human-Directed Social Behaviour** | **Object Directed Play** | **Human Directed Aggressive Behaviour** | **Approach Withdrawal Tendencies** | **Sensitivity to Aversives** |
| Asher et al (2013) | Guide Dog | Yes (no detail) | Yes (no detail) | No | Yes | Yes | No | NA | NA | No |
| Batt et al (2008) | Guide Dog | No | No | No | Yes | NA | No | NA | NA | Yes |
| Foyer et al (2014) | Armed Forces Dog | No | No | No | Yes | NA | NA | Yes | NA | Yes |
| Foyer et al (2016) | Armed Forces Dog | No | No | No | NA | NA | NA | NA | Yes | Yes |
| Gruen et al (2015) | Improvised Explosive Device Detection Dogs | No | No | No | NA | NA | NA | NA | NA | Yes |
| Harvey et al (2016) | Guide Dog | No | Yes | Yes | Yes | No | No | No | Yes | No |
| McGarrity et al (2016) | Odour Detection Dog | Yes | No | Yes | Yes | NA | Yes | NA | Yes | No |
| Sherman et al (2015) | Improvised Explosive Device Detection Dogs. | Yes | No | Yes | NA | NA | NA | NA | NA | Yes |
| Sinn et al (2010) | Military Working Dogs | Yes (separately) | Yes (no statistics) | Yes (separately) | Yes | NA | No | No | NA | No |
| Slabbert et al (1999) | Police Dog | No | No | No | Yes | NA | NA | Yes | MA | Yes and No (different tests) |
| Svartberg (2002) | Working Dog | No | No | No | Yes | NA | Yes | NA | NA | Yes |
| Svobodova et al (2008) | Police Dog | No | No | No | Yes | Yes | NA | NA | NA | Yes |
| Tomkins et al (2011) | Guide Dog | No | No | No | NA | NA | NA | NA | No | NA |
| Weiss (2002) | Guide Dog’s | No | No | No | Yes | No | NA | NA | No | Yes |
| Weiss et al (1997) | Service Dog | No | No | No | NA | No | NA | No | No | No |
| Wilsson et al (1998) | Service Dog | No | No | No | No | Yes | No | No | NA | No |

Supplementary information Table 1. Summary of evaluation of papers included in the review
