# Supplementary material for: A Systematic Review of the Reliability and Validity of Behavioural Tests Used to Assess Behavioural Characteristics Important in Working Dogs
Source: Front Vet Sci. 2018 May 25;5:103. doi: 10.3389/fvets.2018.00103 (PMC5982092; doi:10.3389/fvets.2018.00103)
Supplement: Supplementary file 2 [file Table2.docx]

| **First Author (Date)** | **Aim** | **Working Dog Purpose** | **Sample** | **Behavioural Characteristic** | **Inter-Rater** | **Intra-Rater** | **Test-Retest** | **Predictive Validity** |
| --- | --- | --- | --- | --- | --- | --- | --- | --- |
| Asher et al 2013 | Evaluate the predictive criterion validity of the Puppy Profiling Assessment (PPA) | Guide Dog | 215 females, 250 males, Labradors, Retrievers, Labradors x Retrievers, German Shepherds (and crosses) | Willingness to work (WTW), Human Directed Social Behaviour (HDSB), Object-Directed Play Tendencies (OBP), Sensitivity to Aversives (STA) | Scores were replicated in ~90% of cases (no further statistics presented) | Scores were replicated in ~90% of cases (no further statistics presented) | X | WTW: Retrieve-response to stimuli and ramp tests predicted success in guide dog training (Chi Square tests, *p <* 0.05).  HDSB: Stroking response to assessor was associated with success in guide dog training (Chi Square tests, *p <* 0.05).  ODP: Squirrel-response to stimuli (i.e. playing with toy) did not predict success in guide dog training (Chi Square tests, *p >* 0.05).  STA: Noise response did not predict success in guide dog training (Chi Square tests, *p >* 0.05) |
| Batt et al 2008 | Pilot study to identify whether measures of temperament, lateralization, cortisol activity or colour can determine success | Guide Dog | 27 males, 24 females. Labradors and Retrievers (single breeds) | Willingness to work (WTW), Object-Directed Play Tendencies (OBP), Sensitivity to Aversives (STA) | X | X | X | WTW: Distraction test and passive test predicted guide dog success (Logistic regression model).  ODP: Latency to catch did not significantly predict guide dog success (variable removed from logistic regression model).  STA: Latency to recover from noise significantly predicted guide dog success at 12 months on old (4/5 tests) and 14 months (2/5 test times). |
| Foyer et al (2014) | To investigate whether subjective owner-assessment of dog behaviour, and a questionnaire-based account of environmental events during the first year of life, could predict the outcome of a standardised behaviour test applied at about one and a half years of age to prospective military working dog’s | Swedish Armed Forces Dog’s | 71 German Shepherd’s, Male = 33, Female = 38 | Willingness to Work (WTW), Human Directed Aggressive Behaviour (HDAB), Sensitivity to Aversives (STA) | X | X | X | WTW: Dogs which scored high for C-BARQ category “Trainability” showed a significantly higher success rate in the behaviour test (p < 0.001).  Dogs with higher C-BARQ scores on “Hyperactivity/ restlessness, difficulties in settling down” (p = 0.028), and “Chasing/following light spots” (p = 0.035) were more successful.  HDAB: Dogs that scored high for “Stranger-directed fear” showed a significantly lower success rate (p < 0.05)  STA: Dogs that scored high for “Non-social fear” showed a significantly lower success rate (all P < 0.05). As did dogs that were left longer times at home (2.97 ± 0.32 vs. 2.04 ± 0.33h/day; p = 0.050). |
| Foyer et al (2016) | To evaluate behavioral responses in specific subtests and cortisol responses of candidate dogs | Swedish Armed Forces Dog’s | 85 German Shepherds, Male = 44, Female = 41. 15 – 23 months. | Willingness to Work (WTW), Approach-Withdrawal Tendencies (AWT), Sensitivity to Aversives (STA) | X | X | X | STA: Approved dogs (selected for work) had higher scores on ambivalent fear and overt fear than non-approved dogs.  AWT: Approved dogs had higher scores on active avoidance than non-approved dogs. |
| Gruen et al. (2015) | To apply an open field test to assess sound-induced behaviors in Labrador retrievers who were candidates for detection dog’s | Improvised Explosive Device Detection Dogs | 16 Labrador Retrievers (Intact males = 8, Intact females = 5, and Spayed females = 3, 2 – 4 years | Sensitivity to Aversives (STA) | X | X | X | STA: Fear/anxiety scores, inactive fear/anxiety scores, and global fear/anxiety scores were significantly higher during treatment periods of gunfire and thunder (all p’s < 0.01) |
| Harvey et al (2016) | To design and evaluate test battery for juvenile dog  behavior using a behavioral coding ethogram, for predicting outcomes in a guide dog programme | Guide Dog’s | 93 potential guide dogs, 69 repeated at retest. Female = 52, Male = 41. Breeds specified, predominantly Labrador Retriever crosses | Willingness to Work (WTW), Human Directed Sociability Behaviour (HDSB), Human Directed Aggressive Behaviour (HDAB), Approach-Withdrawal Tendencies (AWT), Object Directed Play (ODP) | Single rater | Intra-Class Coefficient statistics reported. | HDAB: 0.33- 0.49  HDSB: 0.33-0.45  STA: 0.26 -0.66  ODP: 0.39- 0.46  AWT: 0.26-0.61 | WTW: At 5 months, the dogs that later qualified, responded first time to the ‘down’ command from their puppy walker and responded the second or  third time to the novel person for the same command. At 8 months a predictor for success was low scores on distraction.  OBP: At 5 months, the dogs that later qualified scored low on the two five  month component scores (removing teal towel from back, playing with tea towel, barking, lip licking and shaking).  HDSB: At eight months, predictors of guide dog qualification included  not displaying a ‘low’ greeting posture.  AWT: distraction scores were correlated with success or failure as a guide dog. Those distracted more easily were more likely to fail. |
| McGarrity et al (2016) | To test whether two different measurement methods (ratings or codings) would differ in their predictive validity with respect to working-dog selection outcomes | USA Odour Detection Dog’s | 52 dogs Labrador retriever (N = 40), Vizsla (N = 8), and German shorthaired pointer/Labrador mix (N = 4). | Approach Withdrawal Tendencies (AWT), Object Directed Play (ODP), Willingness to Work (WTW)  STA (anxiety score). | AWT: [high inter-observer reliability](https://www.sciencedirect.com/science/article/pii/S0168159116300739#bib0110) on the environment test Average ICC(3,1) = 0.82; L95%C.I. = 0.53, U95%C.I. = 0.92.  WTW: average ICC(3,1) = 0.89 on the search and retrieve (S&R) test. |  | AWT: At 3, 6, 9 and 12 months of age, tests repeated. ICC = 0.35 (environment stability)  STA: ICC =  0.16 (anxiety)  WTW: ICC = 0.28 (hunt drive), 0.11 (search performance) and 0.13 (search aptitude).  ODP: ICC = 0.16 (dominant possession) AND 0.32 (independent possession). | AWT: Environmental stability/confidence significantly predicted selection outcomes.    WTW: Hunt drive was important to selection, but not the dogs’ behaviour across the first year of life relative to the average.  ODP: An individual’s average dominant possession improved chances of selection.  STA: not reported as predictive    There were no differences in predictive validity between the two methods; both ratings and codings correctly classified a high percentage of dogs that were/were not selected for training at 12 months of age (84.6–88.5%).  Rating methods tended to capture behaviour that was more consistent, while coding methods tended to capture behaviour that was more situation-specific. |
| Sherman et al (2015) | To validate an emotional reactivity test (ERT) as  a selection tool for suitable dog’s. | USA Improvised Explosive Device Detection Dogs. | 16 Labrador Retrievers, Male = 8, Female = 8 (3 spayed). Aged 2 – 4 years. Explosive detection dogs | Sensitivity to Aversive (STA) | STA: Inter-rater scores for behavioural tests were moderate to high (visual startle = 0.7; acoustic startle = 0.8; remote control car = 0.7). | X | STA: Tests significantly correlated (*p* < 0.01) (α = 0.89) 10 -12 months later. | STA: The ERT significantly increased salivary cortisol and plasma in the dog’s, suggesting the test can be used to identify dogs with a low threshold for emotional reactivity. |
| Sinn et al 2010 | Aims included evaluating the reliability of  behaviours used in the Lackland MWD behavioral  measurement  and usefulness for predicting the certification outcomes | Military Working Dogs | German Shepherd Dogs (n = 735: female = 125)  Malinois (n = 243, female = 85)  Dutch Shepherd (n = 22, female = 8) | Willingness to work (WTW), Object-Directed Play tendencies (OBP), Human Directed Aggressive Behaviour (HDAB), Sensitivity to Aversives (STA) | WTW: For search activity, search stamina and attention transfer (relevant to willingness to work) inter-rater scores across the three-time points significantly correlated (*p<*.01).  ODP: Inter-rater scores for interest in objects significantly correlated, across three-time points.  HDAB: Inter-rater scores for threat aggression, non-threat bite quality, threat bite quality significantly correlated, across three-time points (*p*<.01).  STA: Inter-rater scores on gun sureness considerably varied over the three-time points, but correlation coefficients were statistically significant. | Stated close agreement; intra-class correlation coefficient, but no supporting statistics presented. | WTW: Spearman’s correlations were significant for comparisons of object focus, sharpness, human focus and search focus between time 1-2, time 1-3, time 2-3 (*ps* < 0.01), with the exception of search focus between time 2-3 (*p*=0.32).  ODP: Inter-rater scores for interest in objects significantly correlated, across three-time points.  HDAB: Threat aggression, non-threat bite quality, threat bite quality significantly correlated across three- time points (*p*<.01).  STA: Gun sureness did not correlate between time 1-2, or time 1-3, only between time 2-3 (*p*< 0.01) | WTW: Search focus and sharpness improved prediction scores of certification outcome in patrol dogs (*p* < 0.01; Wald Chi-square).  ODP: Not reported  HDAB: Not reported  STA: Not reported |
| Slabbert & Odendaal 1999 | Test usefulness of behaviour tests to predict efficient performance in adulthood | South African Police Dog’s | 167 German Shepherd’s | Willingness to work (WTW), Human Directed Aggressive Behaviour (HDAB), Sensitivity to Aversives (STA) | X | X | X | W2W: Retrieve performance (8 & 12 weeks) predicted adult police dog efficiency (*p* < 0.01; statistical test unclear).  HDAB: Aggression (6 & 9 months) predicted adult police dog efficiency.  STA: Gunshot sensitivity did not predict adult police dog efficiency (*p* > 0.05, statistical test unclear). Higher performance on the startle test at 12 and 16 weeks was observed in those who became police dogs, compared to those who did not (*p* < 0.01). |
| Svartberg 2002 | To investigate if there are relationships between personality and performance of dogs in working dog trials | Working Dog’s | German Shepherd dog (male = 875, female = 766) and Belgian Tervuren (male = 191, female = 162) | Human Directed Social Behaviour (HDSB), Object-Directed Play tendencies (OBP), Sensitivity to Aversives (STA) | X | X | X | HDSB: Dogs who scored high on boldness (related to sociability towards strangers) scored significantly higher in working dog trials than those who were scored medium or low in boldness (Kruskal Wallis ANOVA, *p* < 0.05).  ODP: Dogs who scored high on boldness (related to playfulness) scored significantly higher in working dog trials than those who were scored medium or low in boldness (Kruskal Wallis ANOVA, *p* < 0.05).  STA: Dogs who scored high on boldness (related to fearlessness) scored significantly higher in working dog trials than those who were scored medium or low in boldness (Kruskal Wallis ANOVA, *p* < 0.05). |
| Svobodova et al 2008 | Which testable behavioral activities of a puppy  could be used for predicting police and/or military efficiency of the individual. | South African Police Dog’s | 965 German Shepherd’s | Willingness to work (WTW), Human Directed Social Behaviour (HDSB) Sensitivity to Aversives (STA) | X | X | X | WTW: Negotiating obstacles loaded on to the factor for movement. Lower scores on ‘movement’ predicted greater chance of passing police dog test.  HDSB: Decreased scores on ‘factor for movement’ (including behaviour towards a person) predicted increased probability of passing police efficiency test (*p* = 0.02).  STA: Higher scores on ‘factor for responding to noise’ (including, response to distracting stimuli caused by a shovel, response to a distracting noise when left in a room and response to loud distracting stimuli) predicted lower success probabilities for passing the police test (*p* = 0.02). |
| Tomkins et al (2011) | To assess whether temperament tests  including the (i) Passive Test, (ii) Noise Test, (iii) Sudden  Appearance Test, and (iv) Dog Distraction Test, and kennel  behaviour measures  could be used as predictors of success for  dogs/ | Australian Guide Dog’s | 113 potential guide dogs, 13-17 months. Male = 52, Female = 61. Labradors (n = 96), Retrievers (n = 9) Labrador X Retrievers (n = 8) | Approach Withdrawal Tendencies (AWT) | X | X | X | AWT: Approach behaviour in the sudden appearance test did not predict guide dog success. |
| Weiss (2002) | Assess the selection items in an open field test (Activity Level) that are useful for predicting success | USA Guide Dog’s | N = 40 | Willingness to Work (WTW), Human Directed Social Behaviour (HDSB) Sensitivity to Aversives (STA) | X | X | X | WTW: Pearson correlation between the trainer’s assessment of the dog’s potential for service work and whether the dog completed retrieval task was 0.495 (p < .001).  STA: The Pearson correlation for sound sensitivity and the fear rating was 0.427 (p <0.001) and between the pinch test and the trainers submission rating 0.345 (p=0.003).  HDSB: Not reported. |
| Weiss et al (1997) | Evaluate selection tests used with shelter dogs for service work | USA Service Dog’s (from shelters) | 9 dogs. Retriever mixes | Human Directed Aggressive Behaviour (HDAB), Human Directed Social Behaviour (HDSB), Sensitivity to Aversives (STA), Approach Withdrawal Tendencies (AWT) | X | X | X | No predictive ability in terms of selection test predicting performance in training tasks |
| Wilsson & Sundgren 1998 | To investigate if a puppy test could be used to predict  adult usefulness. | Swedish Service Dog’s | 277 female German Shepherd’s, 277 male German Shepherd’s | Willingness to work (WTW), Human Directed Social Behaviour (HDSB), Object-Directed Play tendencies (OBP), Human Directed Aggressive Behaviour (HDAB), Sensitivity to Aversives (STA) | X | X | X | WTW: Generally, not useful for predicting service dog work.  HDSB: Reaction to a stranger at eight-weeks predicted co-operation at maturity in German Shepherd’s.  ODP: Generally, not useful for predicting service dog work. Tug of war at 8 weeks did not correlate with traits at an older age.  HDAB: Generally, not useful for predicting service dog work. Objects visited at 8 weeks correlated with defence drive when older (3-6 yrs) (*p* = 0.02).  STA: Not useful for predicting service dog work. |

Supplementary information Table 2. Summary of evaluation of papers included in the review
