# Supplementary material for: A Systematic Review of the Reliability and Validity of Behavioural Tests Used to Assess Behavioural Characteristics Important in Working Dogs
Source: Front Vet Sci. 2018 May 25;5:103. doi: 10.3389/fvets.2018.00103 (PMC5982092; doi:10.3389/fvets.2018.00103)
Supplement: Supplementary file 3 [file DataSheet1.DOCX]

**Data Sheet 1**. Papers excluded at second pass

1. Barnard, S., Marshall-Pescini, S., Pelosi, A., Passalacqua, C., Prato-Previde, E., & Valsecchi, P. (2017). Breed, sex, and litter effects in 2-month old puppies’ behaviour in a standardised open-field test. *Scientific reports*, *7*(1), 1802.
2. De Meester, R., De Bacquwr, D., Peremans, K., Vermeire, S., Planta, D., Coopman, F., & Audenaert, K. (2008). A preliminary study on the use of the Socially Acceptable Behaviour test as a test for shyness/confidence in the temperament of dogs. *Journal of Veterinary Behaviour*, 3(1), p161-170.
3. De Meester, R., Pluijmakers, J., Vermeire, S., & Laevens, H. (2011). The use of the Socially Acceptable Behaviour test in the study of temperament of dogs. *Journal of Veterinary Behaviour*, 6 (1), p211-224.
4. Diesel, G., Brodbelt, D., & Pfeiffer, D. U. (2008). Reliability of assessment of dogs’ behavioural responses by staff working at a welfare charity in the UK. *Applied Animal Behaviour Science*, *115*(3), 171-181.
5. Dowling-Guyer, S., Marder, A., & D’arpino, S. (2011). Behavioral traits detected in shelter dogs by a behavior evaluation. *Applied animal behaviour science*, *130*(3), 107-114.
6. Foyer, P., Wilsson, E., & Jensen, P. (2016). Levels of maternal care in dogs affect adult offspring temperament. *Scientific reports*, *6*, 19253.
7. Fuchs, T., Gaillard, C., Gebhardt-Henrich, S., Ruefenacht, S., & Steiger, A. (2005). External factors and reproducibility of the behaviour test in German shepherd dogs in Switzerland. *Applied Animal Behaviour Science*, 94 (1), p287-301.
8. Goold, C., & Newberry, R. C. (2017). Modelling personality, plasticity and predictability in shelter dogs. *Royal Society open science*, *4*(9), 170618.
9. Kis, A., Klausz, B., Persa, E., Miklósi, Á., & Gácsi, M. (2014). Timing and presence of an attachment person affect sensitivity of aggression tests in shelter dogs. *Veterinary record*, *174*(8), 196.
10. Klausz, B., Kis, A., Persa, E., Miklosi, A., & Gacsi, M. (2014). A Quick Assessment Tool for Human-Directed Aggression in Pet Dogs. Aggressive Behavior, 40, 178-188.
11. Kubinyi, E., Gosling, S., & Miklósi, Á. (2015). A comparison of rating and coding behavioural traits in dogs. *Acta Biologica Hungarica*, *66*(1), 27-40.
12. Mirkó, E., Dóka, A., & Miklósi, Á. (2013). Association between subjective rating and behaviour coding and the role of experience in making video assessments on the personality of the domestic dog (Canis familiaris). *Applied Animal Behaviour Science*, *149*(1), 45-54.
13. Pérez-Guisado, J., Muñoz-Serrano, A., & López-Rodríguez, R. (2008). Evaluation of the Campbell test and the influence of age, sex, breed, and coat color on puppy behavioral responses. *Canadian Journal of Veterinary Research*, *72*(3), 269.
14. Pérez-Guisado, Joaquín, Rocío Lopez-Rodríguez, and Andrés Muñoz-Serrano. "Heritability of dominant–aggressive behaviour in English Cocker Spaniels." *Applied Animal Behaviour Science* 100, no. 3 (2006): 219-227.
15. Poulsen, A. H., Lisle, A. T., & Phillips, C. J. C. (2010). An evaluation of a behaviour assessment to determine the suitability of shelter dogs for rehoming. *Veterinary medicine international*, *2010*.
16. Riemer, S., Mills, D. S., & Wright, H. (2014). Impulsive for life? The nature of long-term impulsivity in domestic dogs. *Animal cognition*, *17*(3), 815-819.
17. Riemer, S., Müller, C., Virányi, Z., Huber, L., & Range, F. (2013). Choice of conflict resolution strategy is linked to sociability in dog puppies. *Applied animal behaviour science*, *149*(1), 36-44.
18. Riemer, S., Müller, C., Virányi, Z., Huber, L., & Range, F. (2014). The predictive value of early behavioural assessments in pet dogs–a longitudinal study from neonates to adults. *PloS one*, *9*(7), e101237.
19. Roth, L. S., & Jensen, P. (2015). Assessing companion dog behavior in a social setting. *Journal of Veterinary Behavior: Clinical Applications and Research*, *10*(4), 315-323.
20. Sforzini, E., Michelazzi, M., Spada, E., Ricci, C., Carenzi, C., Milani, S., ... & Verga, M. (2009). Evaluation of young and adult dogs' reactivity. *Journal of Veterinary Behavior: Clinical Applications and Research*, *4*(1), 3-10.
21. Svartberg, K. (2005). A comparison of behaviour in test and in everyday life: evidence of three consistent boldness-related personality traits in dogs. *Applied Animal Behaviour Science*, *91*(1), 103-128.
22. Svartberg, K., & Forkman, B. (2002). Personality traits in the domestic dog (Canis familiaris). *Applied Animal Behaviour Science*, *79* (1), 133-155.
23. Svartberg, K., Tapper, I,. Temrin, H., Radesater, T., & Thorman, S. (2005). Consistency of personality traits in dogs. *Animal Behaviour*, *69*(1), 283-291.
24. Valsecchi, P., Barnard, S., Stefanini, C., & Normando, S. (2011). Temperament test for re-homed dogs validated through direct behavioral observation in shelter and home environment. *Journal of Veterinary Behavior: Clinical Applications and Research*, *6*(3), 161-177.
25. van der Borg, J. A., Beerda, B., Ooms, M., de Souza, A. S., van Hagen, M., & Kemp, B. (2010). Evaluation of behaviour testing for human directed aggression in dogs. *Applied Animal Behaviour Science*, *128*(1), 78-90.
26. Wright, H. F., Mills, D. S., & Pollux, P. M. (2012). Behavioural and physiological correlates of impulsivity in the domestic dog (Canis familiaris). *Physiology & behavior*, *105*(3), 676-682.
27. Barnard, S., Marshall-Pescini, S., Passalacqua, C., Beghelli, V., Capra, A., Normando, S., ... & Valsecchi, P. (2016). Does subjective rating reflect behavioural coding? Personality in 2 month-old dog puppies: an open-field test and adjective-based questionnaire. *PloS one*, *11*(3), e0149831.
28. van der Borg, J. A., Netto, W. J., & Planta, D. J. (1991). Behavioural testing of dogs in animal shelters to predict problem behaviour. *Applied Animal Behaviour Science*, *32*(2-3), 237-251.
29. Mongillo, P., Pitteri, E., Adamelli, S., Bonichini, S., Farina, L., & Marinelli, L. (2015). Validation of a selection protocol of dogs involved in animal-assisted intervention. *Journal of Veterinary Behavior: Clinical Applications and Research*, *10*(2), 103-110.
30. Foyer, P., Wilsson, E., Wright, D., & Jensen, P. (2013). Early experiences modulate stress coping in a population of German shepherd dogs. *Applied animal behaviour science*, *146*(1), 79-87.
